# Whole-genome analysis of NDM-producing *Providencia hangzhouensis* associated with recurrent bacteraemia with rapid development of aztreonam-avibactam resistance

**DOI:** 10.1080/22221751.2025.2539193

**Published:** 2025-07-23

**Authors:** Ka Lip Chew, Yuan Qiao, Priscillia Lye, Joey Cruz Cabang, Nur Aisyah Binte Abu Bakar, Ker Xin Tan, Jeanette Teo

**Affiliations:** a Department of Laboratory Medicine, National University Hospital, Singapore; b Nanyang Link, School of Chemistry, Chemical Engineering and Biotechnology (CCEB), Nanyang Technological University, Singapore; c Division of Infectious Diseases, Department of Medicine, Alexandra Hospital, Singapore

**Keywords:** Porin, penicillin-binding protein 3, carbapenem-resistant enterobacterales (CRE), whole-genome sequencing, aztreonam-avibactam

## Abstract

A 60-year-old male with recurrent bacteremia associated with necrotizing pancreatitis was followed over 17 months, during which six clinical isolates of *Providencia* were obtained. The index isolate (R1) was susceptible to aztreonam-avibactam (MIC 4 mg/L), but resistance (MIC >64 mg/L) emerged with isolate B1, collected one month later, and persisted in subsequent isolates. To investigate the genomic and phenotypic evolution of six *Providencia* clinical isolates, focusing on mechanisms underlying the emergence of aztreonam-avibactam resistance. Whole-genome sequencing was performed to determine species identity, phylogenetic relationships, and resistance determinants. Average nucleotide identity (ANI) and in silico DNA–DNA hybridization (DDH) were used to confirm species classification. Comparative genomic analysis with R1 as a reference identified mutations associated with resistance. Structural modelling assessed the functional impact of key mutations. All isolates were sequence type ST44. Phylogenomic analysis revealed the isolates were more closely related to *Providencia hangzhouensis* than *P. rettgeri*, supported by ANI (97%) and DDH (76%). All isolates harboured *bla*_NDM-1_, correlating with carbapenem resistance. Resistant isolates displayed a glycine insertion at position 420 in PBP3 (p.Gly420dup), which structural modelling indicated disrupted aztreonam-avibactam binding. A Gly151Asp substitution in OmpC was also identified, potentially affecting drug permeability. No mutations were observed in other porins (OmpA, OmpD, or OmpW). This study identifies a glycine insertion in PBP3 as a novel mechanism driving aztreonam-avibactam resistance in *Providencia*, supported by structural modelling and additional mutations in OmpC. This study provides evidence of a novel resistance mechanism to aztreonam-avibactam in *Providencia*, driven by a glycine insertion in PBP3 and supported by alterations in OmpC.

## Introduction

Aztreonam-avibactam (ATM-AVI) is a novel β-lactam/β-lactamase inhibitor (BLBLI) combination designed to combat multidrug-resistant Gram-negative bacteria. Aztreonam is stable against hydrolysis by metallo-β-lactamases (MBLs), while avibactam protects aztreonam from serine β-lactamases such as *Klebsiella pneumoniae* carbapenemase (KPC) [1]. This combination holds promise as a treatment option for infections caused by pathogens resistant to other β-lactam/β-lactamase inhibitor combinations or carbapenem-resistant Enterobacterales (CRE) producing serine β-lactamases, MBLs, or both.

While ATM-AVI remains an investigational antibiotic combination [1], it achieved regulatory approval in Europe in April 2024. The European Commission approved aztreonam-avibactam for treating multidrug-resistant infections in patients with limited treatment options (Pfizer, 2024). However, the combination has not yet received FDA approval and is unavailable for clinical use in the United States. Current literature demonstrates high in vitro susceptibility of carbapenem-resistant isolates, including MBL-producing Enterobacterales, to aztreonam-avibactam. Clinical trials have further demonstrated the efficacy and safety of aztreonam-avibactam. The Phase 3 REVISIT study reported clinical cure rates of 76.4% for complicated intra-abdominal infections [2]. These findings underscore its potential as an effective treatment option for infections caused by MBL-producing Enterobacterales [3]. In the absence of widespread availability, clinicians have employed the combination of ceftazidime-avibactam with aztreonam to address infections caused by MBL-producing Enterobacterales [1].

Aztreonam-avibactam minimum inhibitory concentrations (MICs) can now be reliably determined in clinical laboratories using established methods. Studies have shown that MIC values obtained with the MIC Test Strip (MTS) method closely align with those determined by the gold-standard broth microdilution (BMD) method, with an essential agreement (EA) exceeding 90% [4,5].

Penicillin-binding protein 3 (PBP3) is a transpeptidase that facilitates cross-linking of the bacterial cell wall peptidoglycan and serves as a primary target of β-lactam antibiotics. Mutations or structural changes in PBP3 can reduce β-lactam binding and contribute to resistance. Notably, insert modifications in PBP3 have been associated with resistance and treatment failure of aztreonam-avibactam combinations. In particular, four amino acid insertions (YRIN/YRIK) at position 333 of PBP3 have been reported in *Escherichia coli* [6-8], but remain underexplored in other species of Enterobacterales.

The aim of this study is to investigate the genomics of longitudinal *Providencia* isolates from a patient with recurrent carbapenem-resistant bacteraemia, focusing on the rapid development of in vitro aztreonam-avibactam resistance to elucidate its underlying resistance mechanisms.

## Case presentation

A 60-year-old Chinese male with a history of hypertension and hyperlipidemia was admitted in November 2022 for acute pancreatitis, later complicated by necrotizing pancreatitis requiring extensive surgical and intensive care. Following further complications and surgeries, he developed recurrent ventilator-associated pneumonia and empyema, requiring multiple thoracic surgeries, as well as multiple episodes of bacteremia during his admission.

**Figure 1.**
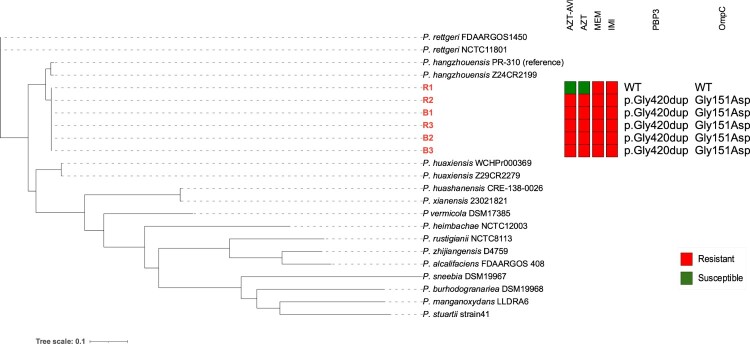
Core-genome SNP phylogeny of clinical *Providencia* isolates. The phylogenetic tree shows the core-genome SNP relationships among clinical *Providencia* isolates, with longitudinal isolates over the study period highlighted in red lettering. The in vitro susceptibilities to aztreonam-avibactam (AZT-AVI), aztreonam (AZT), meropenem (MEM), and imipenem (IMI) are depicted. Metadata, including specimen source and isolation dates, are provided for context. Key mutations in PBP3 and OmpC observed in the aztreonam-avibactam-resistant isolates are listed, with all mutations described relative to the R1 index isolate. These include an in-frame insertion of Gly420dup in PBP3 and the Gly151Asp substitution in OmpC. *Providencia hangzhouensis* PR-310 (GenBank: GCA_029193595.2) was used as the reference genome and the NCBI accessions for other *Providencia* species utilized are provided in the Materials and Methods section.

A carbapenem-resistant NDM-1 producing *Providentia rettgeri* was cultured from a endotracheal tube culture in March 2023 (Isolate R1). He developed his first catheter-related bloodstream infection in April 2023, with cultures from his dialysis catheter yielding an NDM-1-producing *P. rettgeri* (Isolate B1), followed by recurrent episodes of respiratory infections and bacteraemia as with the same organism (Isolates R2 and B2) as detailed in Figure 1.

For each episode of *P. rettgeri* infection, he was treated with intravenous (IV) ceftazidime-avibactam, and aztreonam, with clinical improvement in terms of ventilatory requirements and hemodynamic stability. IV amikacin was also used for short courses during acute deterioration while pending culture sensitivities. After one and a half years, he eventually succumbed with his third episode of *P. rettgeri* bearing NDM-1 bacteraemia in July 2024 (Isolate B3).

## Materials and methods

### Clinical isolates

Longitudinal patient isolates, six in total (R1, R2, R3, B1, B2, B3), were obtained over a 17-month period (Figure 1) and retrospectively analysed. These isolates were identified as *Providencia rettgeri* using Matrix-Assisted Laser Desorption/Ionization Time-of-Flight Mass Spectrometry (MALDI-TOF MS) performed on a Microflex spectrometer (Bruker Daltonics, Bremen, Germany) using the Bruker MALDI Biotyper database (version 3.0).

### Whole-genome-sequencing (WGS), assembly, annotation and resistance gene prediction

Genomic DNA was extracted from overnight plate cultures using DNeasy Blood & Tissue Kits (Qiagen, Hilden, Germany). Illumina NovaSeq 6000 sequencing (Illumina Inc., CA, USA) was used to generate and assemble 150-bp paired-end reads. An average sequencing depth of 150× was achieved for the genomes. Raw reads were trimmed using Trimmomatic v. 0.38 [9] and then assembled with SPAdes version 3.14.0 [10]. BUSCO [11] was used with the bacteria_odb10 database to assess the completeness of genome assemblies. Genome annotation was carried out using Prokka [12]. Plasmid-associated contigs were identified using PlasmidSPAdes [13]. Plasmid replicon types were identified using PlasmidFinder 2.1 (https://cge.food.dtu.dk/services/PlasmidFinder/). To assess plasmid mobility, oriTDB [14] was used to predict origin-of-transfer (oriT) sites, relaxase enzymes, type IV coupling proteins (T4CPs), and type IV secretion system components (T4SS). ABRicate (https://github.com/tseemann/abricate) and Resfinder (http://genepi.food.dtu.dk/resfinder) were used to screen acquired antimicrobial resistance (AMR) determinants. The AMR genes identified in the isolates demonstrated 100% identity to their respective database matches. STs (sequence types) were determined with PubMLST *Providencia* typing database (https://pubmlst.org/bigsdb?db = pubmlst_providencia_seqdef).

Snippy (v4.6.0) (https://github.com/tseemann/snippy) was used to call SNPs and insertions/deletions (indels) between the reference Prokka-annotated GenBank (gbk) file of isolate R1 and subsequent aztreonam-avibactam resistant isolates. Snippy default parameters, with a minimum depth coverage threshold of 10 reads for variant calling was employed.

### PBP3 and ompC structural predictions

Structural predictions of the PBP3 and OmpC were generated using AlphaFold3 (https://alphafoldserver.com) based on the respective protein sequences. The predicted structures were evaluated and aligned using PyMOL (The PyMOL Molecular Graphics System, Version 1.2r3pre, Schrödinger, LLC). Predicted protein stability upon mutation was computed using MAESTROweb [15].

### Phylogenetic analysis

Reference genomes of *Providencia* species were retrieved from GenBank. These included *Providencia alcalifaciens* (GenBank: GCA_002393505.1), *Providencia hangzhouensis* Z24CR2199 (GenBank: GCA_036898125.1), *Providencia hangzhouensis* PR-310 (GenBank: GCA_029193595.2), *Providencia xianensis* (GenBank: GCA_034661195.1), *Providencia huaxiensis* (GenBank: GCF_036898185.1), *Providencia stuartii* (GenBank: GCF_035747985.1), *Providencia vermicola* (GenBank: GCF_020381325.1), *Providencia manganoxydans* (GenBank: GCF_016618195.1), *Providencia zhijiangensis* (GenBank: GCF_030315915.2), and *Providencia rettgeri* isolates 06–1619 (GenBank: GCA_020683065.1) and FDAARGOS 1450 (GenBank: GCA_019048105.1).

Core-genome single nucleotide polymorphisms (SNPs) were detected with the NASP pipeline v.1.0.0 [16] using *P. hangzhouensis* PR-310 (GenBank: GCA_029193595.2) as the reference. Phylogeny was inferred on the concatenated SNP alignment with IQ-TREE 2 software[17]and the tree was visualized with the interactive tree of life (iTOL) [18]. SNP matrix output from NASP was generated using snp-dists (https://github.com/tseemann/snp-dists).

Genome relatedness was assessed using pairwise average nucleotide identity (ANI) and in-silico DNA–DNA hybridization (isDDH) between *Providencia* clinical isolates and the reference genomes (listed above). These analyses were conducted with Pyani (https://github.com/widdowquinn/pyani) and the Genome-to-Genome Distance Calculator (GGDC) formula 2 [19], respectively. A 70.0% isDDH value (Meier-Kolthoff et al. 2022) or a 96% ANI value [20]was used as the threshold to define bacterial species.

### Aztreonam-avibactam and cefiderocol in vitro susceptibility testing

Aztreonam-avibactam susceptibility testing was not available during the patient's initial inpatient period. Retrospective minimum inhibitory concentration (MIC) testing was conducted on stored isolates. Antimicrobial susceptibility testing for aztreonam-avibactam was performed using two methods: gradient diffusion (MIC test range: 0.016/4–256/4 mg/L, MIC Test Strip (MTS), Liofilchem, Italy), broth microdilution EUAZAXF plates (MIC test range: 0.03/4–64/4 mg/L, Sensititre ™ Thermo Fisher Scientific, MA, USA), in accordance with the manufacturers’ instructions. For both methods, the concentration of avibactam was fixed at 4 mg/L.

Minimum inhibitory concentration (MIC) results were interpreted based on the European Committee on Antimicrobial Susceptibility Testing (EUCAST) clinical breakpoints (https://www.eucast.org/clinical_breakpoints), where MIC values of ≤4 mg/L were categorized as sensitive, and MIC values >4 mg/L as resistant. Quality control (QC) was performed using *Escherichia coli* ATCC 25922 and *Klebsiella pneumoniae* ATCC 700603. The expected MIC range for *E. coli* ATCC 25922 was 0.03–0.125 mg/L, with a target MIC of 0.06 mg/L. For *K. pneumoniae* ATCC 700603, the expected MIC range was 0.06–0.5 mg/L, with a target MIC of 0.12–0.25 mg/L. All QC results were within the expected ranges. Phenotypic susceptibility to cefiderocol was determined using the UMIC® Cefiderocol system (Bruker Daltonics, Germany), following the manufacturer’s instructions. Bacterial suspensions were prepared to a 0.5 McFarland standard in cation-adjusted Mueller-Hinton broth (CAMHB) under iron-depleted conditions. The MIC range tested was 0.03–32 mg/L. MIC values were read visually after incubation. Interpretation of results was based on EUCAST clinical breakpoints for Enterobacterales: susceptible ≤2 mg/L; resistant >2 mg/L.

### Data availability

The genome assemblies generated in this study have been deposited in DDBJ/ENA/GenBank under BioProject accession PRJNA1188456, with BioSample and assembly accession numbers as follows: *Providencia* sp. R1 – JBOBHH000000000, R2 – JBOBHL000000000, R3 – JBOBHJ000000000, B1 – JBOBHI000000000, B2 – JBOBHM000000000, and B3 – JBOBHK000000000. Raw sequencing data is available in the NCBI Sequence Read Archive (SRA) under the same BioProject ID.

## Results

### Resistance development of aztreonam-avibactam in longitudinal *providencia c*linical isolates

Six longitudinal patient isolates obtained over a 17-month period were identified as *P. rettgeri* by MALDI-ToF. The index respiratory isolate (R1), collected in March 2023, was carbapenem-resistant but susceptible to aztreonam-avibactam as determined by both MIC test Strip (MTS) and Sensititre. Isolate R1 had an aztreonam-avibactam MIC of 4 mg/L, which was interpreted as susceptible. The aztreonam MIC (without avibactam) was 2 mg/L at the time. However, the next isolate (B1), obtained one month later in April 2023, exhibited no inhibition with either aztreonam alone or in combination with avibactam (MIC > 64 mg/L), a trend observed in all subsequent isolates (Figure 1). All isolates were carbapenem-resistant, with meropenem and imipenem MICs >32 mg/L (Figure 1) (Supplementary Table 1).

### Resistance gene profile and phenotypic correlation in clinical isolates

All six clinical isolates belonged to the same sequence type, ST44, and carried an identical acquired resistance gene profile, including *bla*_NDM-1_, *bla*_PER-4_, *bla*_OXA-10_, *aph(4)-Ia*, *aph(3’)-Ia*, *aac(3)-IV*, *aac(6’)-Ib-cr*, *floR*, *arr-3*, *sul1*, *sul2*, *aadA1*, *ant(2'’)-Ia*, and *catB8**.*** The presence of the NDM-1 gene correlated to phenotypic carbapenem resistance. OXA-10, a class D β-lactamase with weak carbapenemase activity, can confer carbapenem resistance in low outer-membrane permeability backgrounds [21].

### Plasmid context and genetic environment of resistance genes

PlasmidSPAdes analysis revealed that the carbapenemase genes *bla*_NDM-1_, *bla*_PER-4_, *_bla_*_OXA-10_, along with aminoglycoside and other β-lactam resistance determinants, were distributed across three distinct plasmid-derived contigs (Supplementary Fig. 1). These contigs were enriched with insertion sequence (IS) elements. The *bla*_NDM-1_-bearing contig (71,501 bp) contained multiple mobilization-associated genes, including components of the *tra* and *trb* operons, indicating a likely conjugative plasmid backbone. A second contig (27,712 bp) carried both *bla*_OXA-10_ and *bla*_PER-4_, while a third contig (27,330 bp) encoded aminoglycoside-modifying enzymes (Supplementary Fig. 1). No known plasmid replicons were detected on these contigs.

### Whole-genome phylogenetic analysis and isolates identification as *Providencia hangzhouensis*

BUSCO analysis revealed that the genomes showed 100% gene completeness (Supplementary Table 2). The average size of the assembled *Providencia* genomes was approximately 4.6Mbp with an overall G + C content of 40.7 mol%. The genome size and G + C content was comparable to the other members of *Providencia* including *P. hangzhouensis* PR-310 and *P. rettgeri* FDAARGOS1450 (Supplementary Table 3).

The core-genome SNP phylogeny (Figure 1) indicates that the clinical *Providencia* isolates were more closely related to *Providencia hangzhouensis* than to *Providencia rettgeri*. This observation was further supported by species delineation analyses, which reveal that the isolates share approximately 97% average nucleotide identity (ANI) and 76% in silico DNA – DNA hybridization (isDDH) similarity with *P. hangzhouensis* (Supplementary Table 4). In contrast, comparisons with *P. rettgeri* show about 92% ANI and 47% isDDH, underscoring a closer genetic relationship to *P. hangzhouensis* (Supplementary Table 4).

### PBP3 insertion mutation and OmpC Gly151Asp mutation as potential causes of reduced in vitro susceptibility to aztreonam – avibactam

**Figure 2.**
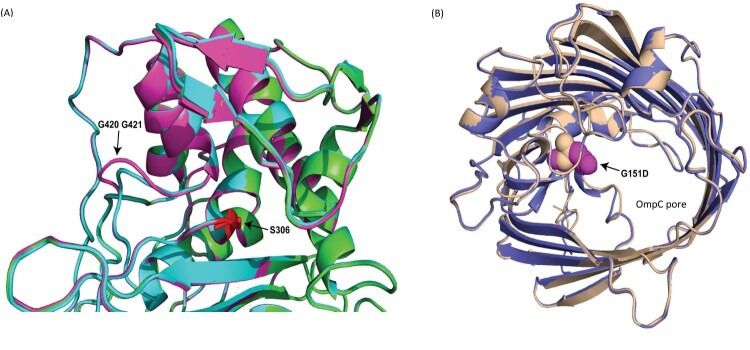
Close-up structural alignment of wild-type *Providencia hangzhouensis* PBP3 and OmpC proteins with mutated proteins from the aztreonam-avibactam resistant isolate (B1). **(A)** Structural comparison of PBP3 proteins. The wild-type *P. hangzhouensis* PBP3 (GenBank accession: CP135052, coding region: 1410452..1412218) is shown in green. The PBP3 from the aztreonam-avibactam susceptible index isolate (R1) is shown in cyan, and the resistant isolate (B1) in magenta. The catalytic residue Ser306 is highlighted in red. An in-frame duplication of Gly420 (Gly420dup) is present in the resistant isolate (B1), resulting in an extended loop near the active site. **(B)** Structural comparison of OmpC proteins. The wild-type *P. hangzhouensis* OmpC (GenBank accession: MEX6121693.1) is shown in wheat. The OmpC variant from the aztreonam-avibactam resistant isolate B1 is shown in blue. The Gly151Asp substitution in the B1 variant is highlighted in magenta and is oriented toward the interior of the pore.

Mutation analysis was conducted by mapping the sequencing reads of sequential aztreonam-avibactam resistant isolates to the index isolate (R1) to identify resistance-associated mutations. Among the SNPs and indels detected in the coding sequences (CDS) of aztreonam-avibactam resistant isolates (Supplementary Table 5), two amino acid mutations were of particular interest: one in PBP3 and the other in OmpC. These mutations were flagged for further investigation due to their potential role in mediating drug resistance[6,22]. In PBP3, aztreonam-avibactam resistant isolates exhibited an in-frame insertion of three nucleotides (+GGG) at nucleotide position 1261, resulting in the addition of a single glycine (Gly) residue at Gly420 (p.Gly420dup) (Figures 1 and 2). Predictive structural analysis revealed that this insertion may cause a structural alteration, leading to a slightly extended loop near the active site (Figure 2). This modification may impair the binding efficacy of aztreonam-avibactam, contributing to the observed resistance.

The Gly151Asp mutation in OmpC is located along the inner surface of the β-barrel, where it introduces a bulkier, negatively charged residue at a structurally relevant site (Figure 2). This substitution could theoretically alter the pore architecture by narrowing the channel diameter and modifying local charge distribution, thereby affecting solute permeability. However, the predicted ΔΔG of −0.488 kcal/mol suggests only mild destabilization of the protein structure. Notably, no mutations were detected in other porins such as OmpA, OmpD, or OmpW.

### PBP3 (p.Gly420dup) and OmpC Gly151Asp mutations do not appear to confer cefiderocol resistance

Porin mutations and target modifications in PBP3 are among the key mechanisms contributing to cefiderocol resistance [23]. For instance, a four-amino-acid insertion (YRIN or YRIK) at position 333 in PBP3 has been associated with reduced cefiderocol susceptibility in Enterobacterales (Sato et al., 2020). Additionally, in Enterobacter spp., alterations in *ompC* and *ompF*, often in combination with AmpC β-lactamases and ESBLs, have been detected as contributors to resistance [24]. Given the presence of the PBP3 (p.Gly420dup) and OmpC Gly151Asp mutations, cefiderocol susceptibility testing was performed to evaluate their potential impact on resistance. All isolates were resistant to cefiderocol, with MICs ranging from 16 to >32 mg/L, suggesting that the PBP3 Gly420dup and OmpC Gly151Asp mutations alone do not confer cefiderocol resistance. However, the absence of established cefiderocol MIC data for *P. hangzhouensis* limits the ability to accurately interpret susceptibility in this species.

## Discussion

Recent studies have identified *Providencia hangzhouensis* as an emerging pathogen, frequently associated with urinary tract infections and often misidentified as *Providencia rettgeri*. Whole-genome sequencing has revealed that *P. hangzhouensis* isolates commonly harbour extended-spectrum β-lactamase (ESBLs) and carbapenem-resistance genes, which can lead to treatment failures in clinical settings. Notably, *bla*_IMP-27_ has been identified as the most prevalent carbapenem-resistance determinant in *P. hangzhouensis*, with a frequency of 46.15% (36 out of 78 isolates) followed by *bla*_NDM-1_ [25]. Our study contributes to the expanding body of evidence on *bla*_NDM_-bearing *Providencia* species implicated in human infections.

Our analysis demonstrated that key antimicrobial resistance genes, including *bla*_NDM-1_, *bla*_PER-4_, and *bla*_OXA-10_, were located on plasmid-associated contigs. Although no known plasmid replicons were detected, the presence of mobilization-associated genes (*tra*, *trb*) and predicted origin-of-transfer (oriT) sites suggests that these contigs may represent plasmids with conjugative potential (Supplementary Fig. 1). Resistance determinants were frequently clustered with insertion sequences and transposases, indicating a mobilizable genetic context. Long-read sequencing should help to resolve plasmid structures and better assess their transfer potential. This plasmid-mediated organization aligns with previous reports in *Providencia* spp., where resistance genes in particular NDM variants are often embedded within complex mobile genetic elements on diverse plasmid backbones [26].

PBP3 insertions are increasingly recognized as contributors to reduced susceptibility to aztreonam-avibactam. Inserts such as YRIK alone or YRIN in combination with acquired AmpC enzymes, particularly CMY-42, have been associated with elevated MICs ranging from 8 to 16 mg/L, compared to the typical *E. coli* susceptible range of 0.03–0.25 mg/L [1,27]. Apart from the YRIN or YRIK insertions after position P333, an unusual β-lactam MIC of 8 μg/ml was reported in an *E. coli* isolate. This isolate carried a TIPY insertion following Tyr344 in PBP3, resulting from gene duplication [28]. In addition to PBP3 mutations, other novel resistance mechanisms have been described. For example, resistance in an *Enterobacter mori* strain was linked to an Arg244Gly substitution in SHV-12 β-lactamase following induced mutagenesis. This mutation disrupted avibactam binding, increasing aztreonam-avibactam MICs to 4 mg/L by altering binding energy and the inhibition constant[29]. Similarly, in vitro selection experiments performed in dual-carbapenemase-producing *Klebsiella pneumoniae* demonstrated that specific substitutions in CMY-16 (Tyr150Ser and Asn346His) significantly reduced avibactam's efficacy, contributing to resistance. These findings highlight the potential for aztreonam-avibactam resistance among MBL – and AmpC-co-producing strains, emphasizing the need for vigilant monitoring and further investigation.

Non-specific porins, such as OmpC and OmpF in *Escherichia coli* (and their corresponding orthologues in other Enterobacterales species), play a significant role in modulating antibiotic susceptibility [30]. In the context of β-lactam resistance, OmpC and OmpF are the primary porins involved, facilitating antibiotic uptake across the bacterial outer membrane. Reduced expression or mutations in these porins decrease membrane permeability, thereby diminishing the effectiveness of β-lactam antibiotics, particularly when coupled with β-lactamase production.

In our study, an in-frame insertion of three nucleotides (+GGG) in the *ftsI* gene resulted in a PBP3 Gly420dup mutation. This mutation appeared in isolates collected after the index isolate R1 and was associated with increased aztreonam-avibactam MICs, suggesting a potential role in resistance. Structural modelling using AlphaFold indicated that the insertion lies within a flexible loop near the catalytic Ser306 residue and may affect drug binding. However, loop regions – especially those with insertions – are challenging to model accurately, and AlphaFold does not account for ligand binding or dynamic conformational shifts [31]. As such, we acknowledge that the structural interpretation remains speculative. Future validation using molecular dynamics simulations or in silico docking will be needed to clarify whether this insertion interferes with aztreonam-avibactam binding [32].

The co-occurrence of a Gly151Asp substitution in OmpC, which could affect outer membrane permeability, may also contribute to the resistance phenotype. However, the ΔΔG for this mutation was modest (−0.488 kcal/mol), suggesting limited structural destabilization. Without permeability assays or additional functional data, its role in resistance remains uncertain. We therefore describe OmpC Gly151Asp as a possible modifier of susceptibility, rather than a primary determinant. As functional validation such as site-directed mutagenesis (SDM) or allelic replacement was not performed, the PBP3 p.Gly420dup and OmpC Gly151Asp mutations are considered putative contributors to reduced aztreonam-avibactam susceptibility, rather than confirmed resistance mechanisms.

Our findings indicate that while the PBP3 p.Gly420dup and OmpC Gly151Asp mutations may contribute to resistance against other β-lactams, they are unlikely to explain the observed cefiderocol resistance. Cefiderocol is a siderophore-cephalosporin, relying on iron transport systems such as CirA, Fiu, and FepA for entry into Gram-negative bacteria. Disruption or loss of function in these iron transporters has been directly linked to cefiderocol resistance in Enterobacterales and *Pseudomonas aeruginosa* [23]. While we examined our *P. hangzhouensis* isolates siderophore receptor genes *cirA* and *fiu*, no truncations or deletions were identified (data not shown). However, in the absence of wild-type MIC distributions specific to *P. hangzhouensis*, it remains challenging to determine the gene polymorphisms that contribute to resistance. Efflux pump overexpression (e.g. RND-type transporters such as AcrAB-TolC) can reduce intracellular antibiotic concentrations, including cefiderocol and may play a role in resistance [23].

In conclusion, this study highlights the genomic and phenotypic complexity of antibiotic resistance in *P. hangzhouensis*, an emerging multidrug-resistant pathogen. The PBP3 p.Gly420dup and OmpC Gly151Asp mutations may contribute to reduced aztreonam-avibactam susceptibility. Continued surveillance and functional validation studies are essential to elucidate resistance mechanisms and inform effective therapeutic strategies against this clinically significant species.

## Supplementary Material

Supplementary Tables_R1.docx
