# Supplementary material for: Whole-genome analysis of NDM-producing Providencia hangzhouensis associated with recurrent bacteraemia with rapid development of aztreonam-avibactam resistance
Source: Emerg Microbes Infect. 2025 Jul 23;14(1):2539193. doi: 10.1080/22221751.2025.2539193 (PMC12329835; doi:10.1080/22221751.2025.2539193)
Supplement: Supplementary Tables_R1.docx [file TEMI_A_2539193_SM5723.docx]

**Supplementary Table 1.** Minimum inhibitory concentrations (MICs) of *Providencia* clinical isolates against a panel of antibiotics. MIC values are shown in mg/L.

| Isolate | Meropenem | Imipenem | Aztreonam | Gentamicin | Tigecycline | Ceftazidime  -avibactam | Colistin | Polymyxin B | Ciprofloxacin | Bactrim |
| --- | --- | --- | --- | --- | --- | --- | --- | --- | --- | --- |
| R1 | >32 | >32 | 2 | >8 | 4 | >32 | >4 | >4 | >2 | >8 |
| B1 | >32 | >32 | >32 | 4 | 8 | >32 | >4 | >4 | >2 | >8 |
| R2 | >32 | >32 | >32 | >8 | 8 | >32 | >4 | >4 | >2 | >8 |
| R3 | >32 | >32 | >32 | >8 | 8 | >32 | >4 | >4 | >2 | >8 |
| B2 | >32 | >32 | >32 | 8 | 4 | >32 | >4 | >4 | >2 | >8 |
| B3 | >32 | >32 | Not done | 8 | Not done | Not done | Not done | Not done | >2 | >8 |

Minimum inhibitory concentration (MIC) results were interpreted based on the European Committee on Antimicrobial Susceptibility Testing (EUCAST) clinical breakpoints (https://www.eucast.org/clinical_breakpoints)

**Supplementary Table 2.**  Genome Assembly and Completeness Metrics for *Providencia* Isolates Based on BUSCO (Benchmarking Universal Single-Copy Orthologs) Analysis Using the 'bacteria_odb10' Dataset

| Isolate | Complete  % | Single-copy % | Duplicated  % | Fragmented  % | Missing  % | Total BUSCO groups searched | Scaffold N50 | Contigs N50 | Percent gaps | Number of scaffolds |
| --- | --- | --- | --- | --- | --- | --- | --- | --- | --- | --- |
| R1 | 100 | 99.2 | 0.8 | 0 | 0 | 124 | 172461 | 172461 | 0 | 77 |
| R2 | 100 | 99.2 | 0.8 | 0 | 0 | 124 | 133433 | 133433 | 0 | 85 |
| R3 | 100 | 99.2 | 0.8 | 0 | 0 | 124 | 133414 | 133414 | 0 | 78 |
| B1 | 100 | 99.2 | 0.8 | 0 | 0 | 124 | 142812 | 142812 | 0 | 67 |
| B2 | 100 | 99.2 | 0.8 | 0 | 0 | 124 | 148249 | 148249 | 0 | 66 |
| B3 | 100 | 99.2 | 0.8 | 0 | 0 | 124 | 154644 | 154644 | 0 | 67 |

**Supplementary Table 3.** Assembly Metrics for *Providencia* Isolates and Reference Genome (FASTA Format)

|  | **R1** | **R2** | **R3** | **B1** | **B2** | **B3** | ***P. hangzhouensis* PR-310 (GCA_029193595.2)** | ***P. rettgeri* FDAARGOS1450 (GCA_019048105.1)** |
| --- | --- | --- | --- | --- | --- | --- | --- | --- |
| Scaffold L50 | 9 | 12 | 13 | 12 | 12 | 11 | 1 | 1 |
| Scaffold N50 | 172461 | 133433 | 133414 | 142812 | 148249 | 154644 | 4515965 | 4492589 |
| Scaffold L90 | 29 | 36 | 36 | 33 | 32 | 32 | 1 | 1 |
| Scaffold N90 | 39164 | 38193 | 39632 | 38193 | 43375 | 39732 | 4515965 | 4492589 |
| Scaffold len_max | 373767 | 372912 | 297249 | 295975 | 371020 | 371020 | 4515965 | 4492589 |
| Scaffold len_min | 536 | 536 | 545 | 542 | 534 | 534 | 2683 | 40804 |
| Scaffold len_mean | 60294 | 54522 | 59050 | 67375 | 69204 | 68146 | 1532674 | 1547314 |
| Scaffold len_median | 20404 | 24904 | 34165 | 37629 | 37418 | 36643 | 79375 | 108549 |
| Scaffold len_std | 86932 | 71275 | 67650 | 71363 | 77382 | 77985 | 2109737 | 2082807 |
| Scaffold num_A | 1374787 | 1376029 | 1370030 | 1342387 | 1358378 | 1357587 | 1369841 | 1380163 |
| Scaffold num_T | 1374754 | 1369169 | 1361703 | 1341133 | 1350357 | 1350355 | 1373273 | 1384584 |
| Scaffold num_C | 965172 | 955332 | 946204 | 927881 | 937135 | 937900 | 927250 | 940589 |
| Scaffold num_G | 927930 | 933908 | 927963 | 902757 | 921640 | 919988 | 927659 | 936606 |
| Scaffold num_N | 0 | 0 | 0 | 0 | 0 | 0 | 0 | 0 |
| Scaffold num_bp | 4642643 | 4634438 | 4605900 | 4514158 | 4567510 | 4565830 | 4598023 | 4641942 |
| Scaffold num_seq | 77 | 85 | 78 | 67 | 66 | 67 | 3 | 3 |
| Scaffold GC content overall | 40.78 | 40.77 | 40.69 | 40.55 | 40.7 | 40.69 | 40.34 | 40.44 |
| Contig L50 | 9 | 12 | 13 | 12 | 12 | 11 | 1 | 1 |
| Contig N50 | 172461 | 133433 | 133414 | 142812 | 148249 | 154644 | 4515965 | 4492589 |
| Contig L90 | 29 | 36 | 36 | 33 | 32 | 32 | 1 | 1 |
| Contig N90 | 39164 | 38193 | 39632 | 38193 | 43375 | 39732 | 4515965 | 4492589 |
| Contig len_max | 373767 | 372912 | 297249 | 295975 | 371020 | 371020 | 4515965 | 4492589 |
| Contig len_min | 536 | 536 | 545 | 542 | 534 | 534 | 2683 | 40804 |
| Contig len_mean | 60294 | 54522 | 59050 | 67375 | 69204 | 68146 | 1532674 | 1547314 |
| Contig len_median | 20404 | 24904 | 34165 | 37629 | 37418 | 36643 | 79375 | 108549 |
| Contig len_std | 86932 | 71275 | 67650 | 71363 | 77382 | 77985 | 2109737 | 2082807 |
| Contig num_bp | 4642643 | 4634438 | 4605900 | 4514158 | 4567510 | 4565830 | 4598023 | 4641942 |
| Contig num_seq | 77 | 85 | 78 | 67 | 66 | 67 | 3 | 3 |
| Number of gaps | 0 | 0 | 0 | 0 | 0 | 0 | 0 | 0 |

**Supplementary Table 4**. Average Nucleotide Identity (ANI) and DNA-DNA hybridization (DDH) of the *Providencia* isolates

| **Isolate** | **GenBank assembly no.** | **ANIb (%)** | | | | | |  | **DDH (%)** | | | | | |
| --- | --- | --- | --- | --- | --- | --- | --- | --- | --- | --- | --- | --- | --- | --- |
|  |  | **R1** | **R3** | **B2** | **B1** | **R2** | **B3** |  | **R1** | **R3** | **B2** | **B1** | **R2** | **B3** |
| **R1** | JBOBHH000000000 | * | 99.9 | 100.0 | 99.9 | 100.0 | 99.9 |  | 100.0 | 100.0 | 100.0 | 100.0 | 100.0 | 100.0 |
| **R3** | JBOBHJ000000000 | 100.0 | 99.9 | 100.0 | 99.9 | * | 99.9 |  | 100.0 | 100.0 | 100.0 | 100.0 | 100.0 | 100.0 |
| **B2** | JBOBHM000000000 | 100.0 | 100.0 | 100.0 | 100.0 | 100.0 | * |  | 100.0 | 100.0 | 100.0 | 100.0 | 100.0 | 100.0 |
| **B1** | JBOBHI000000000 | 100.0 | * | 100.0 | 99.9 | 100.0 | 99.9 |  | 100.0 | 100.0 | 100.0 | 100.0 | 100.0 | 100.0 |
| **R2** | JBOBHL000000000 | 100.0 | 99.9 | * | 99.9 | 100.0 | 99.9 |  | 100.0 | 100.0 | 100.0 | 100.0 | 100.0 | 100.0 |
| **B3** | JBOBHK000000000 | 100.0 | 100.0 | 100.0 | * | 100.0 | 100.0 |  | 100.0 | 100.0 | 100.0 | 100.0 | 100.0 | 100.0 |
| ***P. hangzhouensis* PR-310** | GCA_029193595.2 | 97.0 | 97.0 | 97.0 | 97.0 | 97.0 | 97.0 |  | 75.9 | 75.9 | 75.9 | 75.9 | 75.8 | 75.9 |
| ***P. hangzhouensis* Z24CR2199** | GCA_036898125.1 | 96.6 | 96.6 | 96.5 | 96.5 | 96.5 | 96.5 |  | 75.3 | 75.9 | 75.2 | 75.3 | 75.3 | 75.2 |
| ***P. rettgeri* FDAARGOS1450** | GCA_019048105.1 | 91.6 | 91.6 | 91.6 | 91.6 | 91.6 | 91.6 |  | 47.1 | 47.1 | 47.1 | 47.1 | 47.1 | 47.2 |
| ***P. rettgeri* NCTC11801** | GCA_900455085.1 | 91.6 | 91.6 | 91.6 | 91.6 | 91.6 | 91.6 |  | 47.0 | 47.1 | 47.1 | 47.1 | 47.0 | 47.1 |
| ***P. huaxiensis* WCHPr000369** | GCA_002843235.3 | 91.7 | 91.7 | 91.7 | 91.7 | 91.7 | 91.7 |  | 46.7 | 46.8 | 46.8 | 46.8 | 46.7 | 46.8 |
| ***P. huashanensis* CRE-138-0026** | GCA_030580945.1 | 83.3 | 82.9 | 83.3 | 83.3 | 83.2 | 83.3 |  | 27.1 | 27.1 | 27.1 | 27.1 | 27.0 | 27.1 |
| ***P. xianensis* 23021821** | GCA_034661195.1 | 82.3 | 82.3 | 82.3 | 82.3 | 82.3 | 82.3 |  | 27.1 | 25.7 | 25.7 | 25.7 | 25.7 | 25.7 |
| ***P. vermicola* DSM17385** | GCA_020381325.1 | 80.8 | 80.8 | 80.8 | 80.8 | 80.8 | 80.8 |  | 25.7 | 23.7 | 23.7 | 23.7 | 23.7 | 23.7 |
| ***P. heimbachae* NCTC12003** | GCA_900475855 | 78.6 | 78.6 | 78.6 | 78.5 | 78.6 | 78.6 |  | 23.7 | 22.0 | 22.0 | 22.0 | 22.0 | 22.0 |
| ***P. manganoxydans* LLDRA6** | GCA_016618195.1 | 76.7 | 76.7 | 76.7 | 76.6 | 76.6 | 76.6 |  | 22.0 | 21.6 | 21.5 | 21.5 | 21.5 | 21.5 |
| ***P. zhijiangensis* D4759** | GCA_000314895.2 | 76.7 | 76.7 | 76.7 | 76.6 | 76.7 | 76.7 |  | 21.5 | 21.5 | 21.5 | 21.5 | 21.5 | 21.5 |
| ***P. sneebia* DSM 19967** | GCA_030315915 | 77.8 | 77.8 | 77.8 | 77.7 | 77.7 | 77.8 |  | 21.5 | 21.5 | 21.4 | 21.4 | 21.4 | 21.4 |
| ***P. stuartii* strain 41** | GCA_000314855.2 | 76.8 | 76.8 | 76.8 | 76.8 | 76.8 | 76.8 |  | 21.4 | 21.5 | 21.4 | 21.4 | 21.4 | 21.4 |
| ***P. burhodogranariea* DSM 19968** | GCA_035747985.1 | 77.0 | 77.0 | 77.0 | 76.8 | 76.9 | 76.8 |  | 21.4 | 21.4 | 21.4 | 21.4 | 21.3 | 21.2 |
| ***P. rustigianii* NCTC8113** | GCA_900637755 | 77.6 | 77.6 | 77.6 | 77.5 | 77.5 | 77.5 |  | 21.4 | 21.2 | 21.2 | 21.1 | 21.1 | 21.2 |
| ***P. alcalifaciens* FDAARGOS_408** | GCA_002393505.1 | 77.2 | 77.2 | 77.2 | 77.2 | 77.2 | 77.2 |  | 21.2 | 21.3 | 21.2 | 21.2 | 21.2 | 21.2 |

| **Supplementary Table 5.** Single Nucleotide Polymorphisms (SNPs) and Insertions/Deletions (INDELs)  Identified in Clinical Isolates Relative to Reference Isolate R1 Using Snippy | | | | | | | | | |
| --- | --- | --- | --- | --- | --- | --- | --- | --- | --- |
| **IsolateR2** | | |  |  |  |  |  |  |  |
| S/N | TYPE | FTYPE | EFFECT | GENE | PRODUCT | CATEGORY |  |  |  |
| 1 | snp | CDS | missense_variant c.1348T>A p.Cys450Ser | vgrG | type VI secretion system tip protein VgrG | Stress Response and Regulatory Functions |  |  |  |
| 2 | snp | CDS | missense_variant c.46G>A p.Ala16Thr | dpiA | two-component response regulator DpiA | Stress Response and Regulatory Functions |  |  |  |
| 3 | snp | CDS | missense_variant c.688C>A p.Leu230Ile | narX_1 | nitrate/nitrite two-component system sensor histidine kinase NarX | Stress Response and Regulatory Functions |  |  |  |
| 4 | snp | CDS | missense_variant c.219G>A p.Met73Ile | MurR/RpiR | MurR/RpiR family transcriptional regulator | Stress Response and Regulatory Functions |  |  |  |
| 5 | snp | CDS | missense_variant c.541C>T p.Arg181Cys | crp | cAMP-activated global transcriptional regulator CRP | Stress Response and Regulatory Functions |  |  |  |
| 6 | snp | CDS | missense_variant c.278C>A p.Thr93Lys | ahpD | alkylhydroperoxidase domain protein | Stress Response and Regulatory Functions |  |  |  |
| 7 | snp | CDS | stop_gained c.193G>T p.Glu65* | MAPEG | MAPEG family protein | Resistance Mechanisms and Cell Survival |  |  |  |
| 8 | snp | CDS | missense_variant c.568T>C p.Phe190Leu | macB_1 | macrolide ABC transporter ATP-binding protein/permease MacB | Resistance Mechanisms and Cell Survival |  |  |  |
| 9 | snp | CDS | missense_variant c.821G>A p.Ser274Asn | dacA | D-alanyl-D-alanine carboxypeptidase DacA | Resistance Mechanisms and Cell Survival |  |  |  |
| 10 | snp | CDS | stop_gained c.1652C>G p.Ser551* | cas3 | CRISPR-associated nuclease/helicase Cas3 subtypeI-F/YPEST | Resistance Mechanisms and Cell Survival |  |  |  |
| 11 | snp | CDS | missense_variant c.172G>A p.Ala58Thr | arsB_1 | arsenite/antimonite:H(+) antiporter ArsB | Resistance Mechanisms and Cell Survival |  |  |  |
| 12 | snp | CDS | synonymous_variant c.321C>T p.Ala107Ala | arsD_2 | Arsenical resistance operon trans-acting repressor ArsD | Resistance Mechanisms and Cell Survival |  |  |  |
| 13 | snp | CDS | synonymous_variant c.876G>A p.Arg292Arg | rdgC_2 | Recombination-associated protein RdgC | Protein Synthesis and Genetic Information Processing |  |  |  |
| 14 | snp | CDS | missense_variant c.670G>A p.Gly224Ser | recD | exodeoxyribonuclease V subunit alpha | Protein Synthesis and Genetic Information Processing |  |  |  |
| 15 | snp | CDS | missense_variant c.634A>G p.Thr212Ala | dnaX | DNA polymerase III subunit gamma/tau | Protein Synthesis and Genetic Information Processing |  |  |  |
| 16 | snp | CDS | missense_variant c.1459G>A p.Gly487Arg | mukB | chromosome partition protein MukB | Protein Synthesis and Genetic Information Processing |  |  |  |
| 17 | snp | CDS | synonymous_variant c.459C>T p.Val153Val | rpsC | 30S ribosomal protein S3 | Protein Synthesis and Genetic Information Processing |  |  |  |
| 18 | snp | CDS | missense_variant c.379A>C p.Thr127Pro | xylB | xylulokinase | Metabolism |  |  |  |
| 19 | snp | CDS | missense_variant c.418A>G p.Thr140Ala | btuD_3 | Vitamin B12 import ATP-binding protein BtuD | Metabolism |  |  |  |
| 20 | snp | CDS | stop_gained c.481C>T p.Gln161* | torA | trimethylamine-N-oxide reductase TorA | Metabolism |  |  |  |
| 21 | snp | CDS | missense_variant c.172A>G p.Thr58Ala | pyk | pyruvate kinase | Metabolism |  |  |  |
| 22 | snp | CDS | missense_variant c.290T>C p.Val97Ala | pitA | inorganic phosphate transporter PitA | Metabolism |  |  |  |
| 23 | snp | CDS | missense_variant c.26G>A p.Arg9His | ribA | GTP cyclohydrolase II | Metabolism |  |  |  |
| 24 | snp | CDS | missense_variant c.1243G>A p.Asp415Asn | gadB | glutamate decarboxylase | Metabolism |  |  |  |
| 25 | snp | CDS | missense_variant c.466T>C p.Ser156Pro | purT | formate-dependent phosphoribosylglycinamide formyltransferase | Metabolism |  |  |  |
| 26 | snp | CDS | missense_variant c.76C>T p.Leu26Phe | caiC | crotonobetaine/carnitine-CoA ligase | Metabolism |  |  |  |
| 27 | snp | CDS | synonymous_variant c.270G>A p.Leu90Leu | dadX | catabolic alanine racemase DadX | Metabolism |  |  |  |
| 28 | snp | CDS | missense_variant c.142A>G p.Asn48Asp | dcuA_2 | anaerobic C4-dicarboxylate transporter DcuA | Metabolism |  |  |  |
| 29 | snp | CDS | missense_variant c.101G>A p.Gly34Glu | aap | amino acid permease | Metabolism |  |  |  |
| 30 | snp | CDS | missense_variant c.476T>C p.Phe159Ser | kdc | alpha-keto acid decarboxylase family protein | Metabolism |  |  |  |
| 31 | snp | CDS | missense_variant c.50C>T p.Ala17Val | accA_2 | acetyl-CoA carboxylase carboxyl transferase subunit alpha | Metabolism |  |  |  |
| 32 | snp | CDS | missense_variant c.266T>C p.Leu89Pro | yfdR | 5'-deoxynucleotidase | Metabolism |  |  |  |
| 33 | snp | CDS | missense_variant c.91T>A p.Ser31Thr | shlB/fhaC/hecB | ShlB/FhaC/HecB family hemolysin secretion/activation protein | Cellular Structure and Transport |  |  |  |
| 34 | snp | CDS | missense_variant c.452G>A p.Gly151Asp | ompC | porin OmpC | Cellular Structure and Transport |  |  |  |
| 35 | ins | CDS | conservative_inframe_insertion c.1258_1260dupGGG p.Gly420dup | ftsI | peptidoglycan glycosyltransferase FtsI | Cellular Structure and Transport |  |  |  |
| 36 | snp | CDS | missense_variant c.83G>A p.Ser28Asn | lptF | LPS export ABC transporter permease LptF | Cellular Structure and Transport |  |  |  |
| 37 | snp | CDS | missense_variant c.308T>A p.Leu103Gln | ynaI | low conductance mechanosensitive channel YnaI | Cellular Structure and Transport |  |  |  |
| 38 | snp | CDS | missense_variant c.782A>C p.Lys261Thr | FliC/FljB | FliC/FljB family flagellin | Cellular Structure and Transport |  |  |  |
| 39 | snp | CDS | missense_variant c.652A>G p.Ser218Gly | FliC/FljB | FliC/FljB family flagellin | Cellular Structure and Transport |  |  |  |
| 40 | snp | CDS | missense_variant c.632G>A p.Arg211Gln | ABC-F family ATP-binding cassette | ABC-F family ATP-binding cassette domain-containing protein | Cellular Structure and Transport |  |  |  |
| 41 | del | CDS | frameshift_variant c.309delC p.Gly104fs | ABC transporter ATP-binding cassette | ABC transporter ATP-binding protein | Cellular Structure and Transport |  |  |  |
|  |  |  |  |  |  |  |  |  |  |
| **Isolate R3** | | |  |  |  |  |  |  |  |
| S/N | TYPE | FTYPE | EFFECT | GENE | PRODUCT | CATEGORY |  |  |  |
| 1 | snp | CDS | missense_variant c.278C>A p.Thr93Lys | alkylhydroperoxidase domain protein | alkylhydroperoxidase domain protein | Stress Response and Regulatory Functions |  |  |  |
| 2 | snp | CDS | missense_variant c.814G>A p.Val272Ile | sufB | Fe-S cluster assembly protein SufB | Stress Response and Regulatory Functions |  |  |  |
| 3 | snp | CDS | missense_variant c.219G>A p.Met73Ile | MurR/RpiR | MurR/RpiR family transcriptional regulator | Stress Response and Regulatory Functions |  |  |  |
| 4 | snp | CDS | missense_variant c.688C>A p.Leu230Ile | narX_1 | nitrate/nitrite two-component system sensor histidine kinase NarX | Stress Response and Regulatory Functions |  |  |  |
| 5 | snp | CDS | missense_variant c.46G>A p.Ala16Thr | dpiA | two-component response regulator DpiA | Stress Response and Regulatory Functions |  |  |  |
| 6 | snp | CDS | missense_variant c.1348T>A p.Cys450Ser | vgrG | type VI secretion system tip protein VgrG | Stress Response and Regulatory Functions |  |  |  |
| 7 | snp | CDS | synonymous_variant c.321C>T p.Ala107Ala | arsD_2 | Arsenical resistance operon trans-acting repressor ArsD | Resistance Mechanisms and Cell Survival |  |  |  |
| 8 | snp | CDS | missense_variant c.172G>A p.Ala58Thr | arsB_1 | arsenite/antimonite:H(+) antiporter ArsB | Resistance Mechanisms and Cell Survival |  |  |  |
| 9 | snp | CDS | stop_gained c.1652C>G p.Ser551* | cas3 | CRISPR-associated nuclease/helicase Cas3 subtypeI-F/YPEST | Resistance Mechanisms and Cell Survival |  |  |  |
| 10 | snp | CDS | missense_variant c.821G>A p.Ser274Asn | dacA | D-alanyl-D-alanine carboxypeptidase DacA | Resistance Mechanisms and Cell Survival |  |  |  |
| 11 | snp | CDS | missense_variant c.568T>C p.Phe190Leu | macB_1 | macrolide ABC transporter ATP-binding protein/permease MacB | Resistance Mechanisms and Cell Survival |  |  |  |
| 12 | snp | CDS | missense_variant c.20C>T p.Pro7Leu | yceD | 23S rRNA accumulation protein YceD | Protein Synthesis and Genetic Information Processing |  |  |  |
| 13 | snp | CDS | synonymous_variant c.459C>T p.Val153Val | rpsC | 30S ribosomal protein S3 | Protein Synthesis and Genetic Information Processing |  |  |  |
| 14 | snp | CDS | missense_variant c.1459G>A p.Gly487Arg | mukB | chromosome partition protein MukB | Protein Synthesis and Genetic Information Processing |  |  |  |
| 15 | snp | CDS | missense_variant c.634A>G p.Thr212Ala | dnaX | DNA polymerase III subunit gamma/tau | Protein Synthesis and Genetic Information Processing |  |  |  |
| 16 | snp | CDS | missense_variant c.623A>T p.Asn208Ile | rpoA | DNA-directed RNA polymerase subunit alpha | Protein Synthesis and Genetic Information Processing |  |  |  |
| 17 | snp | CDS | missense_variant c.670G>A p.Gly224Ser | recD | exodeoxyribonuclease V subunit alpha | Protein Synthesis and Genetic Information Processing |  |  |  |
| 18 | snp | CDS | missense_variant c.1085C>T p.Pro362Leu | pcnB | polynucleotide adenylyltransferase PcnB | Protein Synthesis and Genetic Information Processing |  |  |  |
| 19 | snp | CDS | synonymous_variant c.876G>A p.Arg292Arg | rdgC_2 | Recombination-associated protein RdgC | Protein Synthesis and Genetic Information Processing |  |  |  |
| 20 | snp | CDS | missense_variant c.1581A>T p.Leu527Phe | rtcR | RNA repair transcriptional activator RtcR | Protein Synthesis and Genetic Information Processing |  |  |  |
| 21 | snp | CDS | missense_variant c.172A>G p.Thr58Ala | pyk | pyruvate kinase | Metabolism |  |  |  |
| 22 | snp | CDS | missense_variant c.379A>C p.Thr127Pro | xylB | xylulokinase | Metabolism |  |  |  |
| 23 | snp | CDS | stop_gained c.481C>T p.Gln161* | torA | trimethylamine-N-oxide reductase TorA | Metabolism |  |  |  |
| 24 | snp | CDS | synonymous_variant c.270G>A p.Leu90Leu | dadX | catabolic alanine racemase DadX | Metabolism |  |  |  |
| 25 | snp | CDS | missense_variant c.266T>C p.Leu89Pro | yfdR | 5'-deoxynucleotidase | Metabolism |  |  |  |
| 26 | snp | CDS | missense_variant c.476T>C p.Phe159Ser | alpha-keto acid decarboxylase family protein | alpha-keto acid decarboxylase family protein | Metabolism |  |  |  |
| 27 | snp | CDS | missense_variant c.101G>A p.Gly34Glu | amino acid permease | amino acid permease | Metabolism |  |  |  |
| 28 | snp | CDS | missense_variant c.142A>G p.Asn48Asp | dcuA_2 | anaerobic C4-dicarboxylate transporter DcuA | Metabolism |  |  |  |
| 29 | del | CDS | frameshift_variant c.250delG p.Ala84fs | fabF_2 | beta-ketoacyl-ACP synthase II | Metabolism |  |  |  |
| 30 | snp | CDS | missense_variant c.1129G>A p.Glu377Lys | spoT | bifunctional GTP diphosphokinase/guanosine-3',5'-bis pyrophosphate 3'-pyrophosphohydrolase | Metabolism |  |  |  |
| 31 | snp | CDS | missense_variant c.76C>T p.Leu26Phe | caiC | crotonobetaine/carnitine-CoA ligase | Metabolism |  |  |  |
| 32 | snp | CDS | missense_variant c.466T>C p.Ser156Pro | purT | formate-dependent phosphoribosylglycinamide formyltransferase | Metabolism |  |  |  |
| 33 | snp | CDS | missense_variant c.1243G>A p.Asp415Asn | gadB | glutamate decarboxylase | Metabolism |  |  |  |
| 34 | snp | CDS | missense_variant c.290T>C p.Val97Ala | pitA | inorganic phosphate transporter PitA | Metabolism |  |  |  |
| 35 | del | CDS | frameshift_variant c.268delG p.Ala90fs | ptrA | pitrilysin | Metabolism |  |  |  |
| 36 | snp | CDS | missense_variant c.418A>G p.Thr140Ala | btuD_3 | Vitamin B12 import ATP-binding protein BtuD | Metabolism |  |  |  |
| 37 | snp | CDS | missense_variant c.782A>C p.Lys261Thr | FliC/FljB | FliC/FljB family flagellin | Cellular Structure and Transport |  |  |  |
| 38 | snp | CDS | missense_variant c.652A>G p.Ser218Gly | FliC/FljB | FliC/FljB family flagellin | Cellular Structure and Transport |  |  |  |
| 39 | snp | CDS | missense_variant c.308T>A p.Leu103Gln | ynaI | low conductance mechanosensitive channel YnaI | Cellular Structure and Transport |  |  |  |
| 40 | snp | CDS | missense_variant c.83G>A p.Ser28Asn | lptF | LPS export ABC transporter permease LptF | Cellular Structure and Transport |  |  |  |
| 41 | ins | CDS | p.Gly420dup | ftsI | peptidoglycan glycosyltransferase FtsI | Cellular Structure and Transport |  |  |  |
| 42 | snp | CDS | missense_variant c.452G>A p.Gly151Asp | ompC | porin OmpC | Cellular Structure and Transport |  |  |  |
| 43 | snp | CDS | missense_variant c.91T>A p.Ser31Thr | ShlB/FhaC/HecB | ShlB/FhaC/HecB family hemolysin secretion/activation protein | Cellular Structure and Transport |  |  |  |
|  |  |  |  |  |  |  |  |  |  |
| **Isolate B1** | | |  |  |  |  |  |  |  |
| S/N | TYPE | FTYPE | EFFECT | GENE | PRODUCT | CATEGORY |  |  |  |
| 1 | snp | CDS | missense_variant c.278C>A p.Thr93Lys | alkylhydroperoxidase domain protein | alkylhydroperoxidase domain protein | Stress Response and Regulatory Functions |  |  |  |
| 2 | snp | CDS | missense_variant c.308T>A p.Leu103Gln | ynaI | low conductance mechanosensitive channel YnaI | Stress Response and Regulatory Functions |  |  |  |
| 3 | snp | CDS | missense_variant c.46G>A p.Ala16Thr | dpiA | two-component response regulator DpiA | Stress Response and Regulatory Functions |  |  |  |
| 4 | snp | CDS | synonymous_variant c.321C>T p.Ala107Ala | arsD_2 | Arsenical resistance operon trans-acting repressor ArsD | Resistance Mechanisms and Cell Survival |  |  |  |
| 5 | snp | CDS | missense_variant c.172G>A p.Ala58Thr | arsB_1 | arsenite/antimonite:H(+) antiporter ArsB | Resistance Mechanisms and Cell Survival |  |  |  |
| 6 | snp | CDS | stop_gained c.1652C>G p.Ser551* | cas3 | CRISPR-associated nuclease/helicase Cas3 subtypeI-F/YPEST | Resistance Mechanisms and Cell Survival |  |  |  |
| 7 | snp | CDS | missense_variant c.568T>C p.Phe190Leu | macB_1 | macrolide ABC transporter ATP-binding protein/permease MacB | Resistance Mechanisms and Cell Survival |  |  |  |
| 8 | snp | CDS | missense_variant c.688C>A p.Leu230Ile | narX_1 | nitrate/nitrite two-component system sensor histidine kinase NarX | Resistance Mechanisms and Cell Survival |  |  |  |
| 9 | snp | CDS | missense_variant c.91T>A p.Ser31Thr | ShlB/FhaC/HecB | ShlB/FhaC/HecB family hemolysin secretion/activation protein | Resistance Mechanisms and Cell Survival |  |  |  |
| 10 | snp | CDS | missense_variant c.1348T>A p.Cys450Ser | vgrG | type VI secretion system tip protein VgrG | Resistance Mechanisms and Cell Survival |  |  |  |
| 11 | snp | CDS | synonymous_variant c.459C>T p.Val153Val | rpsC | 30S ribosomal protein S3 | Protein Synthesis and Genetic Information Processing |  |  |  |
| 12 | snp | CDS | missense_variant c.634A>G p.Thr212Ala | dnaX | DNA polymerase III subunit gamma/tau | Protein Synthesis and Genetic Information Processing |  |  |  |
| 13 | snp | CDS | missense_variant c.670G>A p.Gly224Ser | recD | exodeoxyribonuclease V subunit alpha | Protein Synthesis and Genetic Information Processing |  |  |  |
| 14 | snp | CDS | missense_variant c.219G>A p.Met73Ile | MurR/RpiR | MurR/RpiR family transcriptional regulator | Protein Synthesis and Genetic Information Processing |  |  |  |
| 15 | snp | CDS | missense_variant c.266T>C p.Leu89Pro | yfdR | 5'-deoxynucleotidase | Metabolism |  |  |  |
| 16 | snp | CDS | missense_variant c.476T>C p.Phe159Ser | alpha-keto acid decarboxylase family protein | alpha-keto acid decarboxylase family protein | Metabolism |  |  |  |
| 17 | snp | CDS | missense_variant c.101G>A p.Gly34Glu | amino acid permease | amino acid permease | Metabolism |  |  |  |
| 18 | snp | CDS | synonymous_variant c.270G>A p.Leu90Leu | dadX | catabolic alanine racemase DadX | Metabolism |  |  |  |
| 19 | snp | CDS | missense_variant c.76C>T p.Leu26Phe | caiC | crotonobetaine/carnitine-CoA ligase | Metabolism |  |  |  |
| 20 | snp | CDS | missense_variant c.821G>A p.Ser274Asn | dacA | D-alanyl-D-alanine carboxypeptidase DacA | Metabolism |  |  |  |
| 21 | snp | CDS | missense_variant c.466T>C p.Ser156Pro | purT | formate-dependent phosphoribosylglycinamide formyltransferase | Metabolism |  |  |  |
| 22 | snp | CDS | missense_variant c.1243G>A p.Asp415Asn | gadB | glutamate decarboxylase | Metabolism |  |  |  |
| 23 | snp | CDS | missense_variant c.172A>G p.Thr58Ala | pyk | pyruvate kinase | Metabolism |  |  |  |
| 24 | snp | CDS | stop_gained c.481C>T p.Gln161* | torA | trimethylamine-N-oxide reductase TorA | Metabolism |  |  |  |
| 25 | snp | CDS | missense_variant c.418A>G p.Thr140Ala | btuD_3 | Vitamin B12 import ATP-binding protein BtuD | Metabolism |  |  |  |
| 26 | snp | CDS | missense_variant c.379A>C p.Thr127Pro | xylB | xylulokinase | Metabolism |  |  |  |
| 27 | snp | CDS | missense_variant c.142A>G p.Asn48Asp | dcuA_2 | anaerobic C4-dicarboxylate transporter DcuA | Cellular Structure and Transport |  |  |  |
| 28 | snp | CDS | missense_variant c.1459G>A p.Gly487Arg | mukB | chromosome partition protein MukB | Cellular Structure and Transport |  |  |  |
| 29 | snp | CDS | missense_variant c.782A>C p.Lys261Thr | FliC/FljB | FliC/FljB family flagellin | Cellular Structure and Transport |  |  |  |
| 30 | snp | CDS | missense_variant c.652A>G p.Ser218Gly | FliC/FljB | FliC/FljB family flagellin | Cellular Structure and Transport |  |  |  |
| 31 | snp | CDS | missense_variant c.290T>C p.Val97Ala | pitA | inorganic phosphate transporter PitA | Cellular Structure and Transport |  |  |  |
| 32 | snp | CDS | missense_variant c.83G>A p.Ser28Asn | lptF | LPS export ABC transporter permease LptF | Cellular Structure and Transport |  |  |  |
| 33 | ins | CDS | conservative_inframe_insertion c.1258_1260dupGGG p.Gly420dup | ftsI | peptidoglycan glycosyltransferase FtsI | Cellular Structure and Transport |  |  |  |
| 34 | snp | CDS | missense_variant c.452G>A p.Gly151Asp | ompC | porin OmpC | Cellular Structure and Transport |  |  |  |
|  |  |  |  |  |  |  |  |  |  |
|  |  |  |  |  |  |  |  |  |  |
| **Isolate B2** | | | |  |  |  |  |  |  |
| S/N | TYPE | FTYPE | EFFECT | GENE | PRODUCT | CATEGORY |  |  |  |
| 1 | snp | CDS | missense_variant c.229C>T p.Leu77Phe | YgiW/YdeI | YgiW/YdeI family stress tolerance OB fold protein | Stress Response and Regulatory Functions |  |  |  |
| 2 | snp | CDS | missense_variant c.46G>A p.Ala16Thr | dpiA | two-component response regulator DpiA | Stress Response and Regulatory Functions |  |  |  |
| 3 | snp | CDS | missense_variant c.308T>A p.Leu103Gln | ynaI | low conductance mechanosensitive channel YnaI | Stress Response and Regulatory Functions |  |  |  |
| 4 | del | CDS | frameshift_variant c.96delG p.Trp32fs | glutathionylspermidine synthase family protein | glutathionylspermidine synthase family protein | Stress Response and Regulatory Functions |  |  |  |
| 5 | snp | CDS | missense_variant c.278C>A p.Thr93Lys | alkylhydroperoxidase domain protein | alkylhydroperoxidase domain protein | Stress Response and Regulatory Functions |  |  |  |
| 6 | snp | CDS | synonymous_variant c.180G>C p.Gly60Gly | AAA family ATPase | AAA family ATPase | Stress Response and Regulatory Functions |  |  |  |
| 7 | snp | CDS | missense_variant c.1348T>A p.Cys450Ser | vgrG | type VI secretion system tip protein VgrG | Resistance Mechanisms and Cell Survival |  |  |  |
| 8 | snp | CDS | missense_variant c.91T>A p.Ser31Thr | ShlB/FhaC/HecB | ShlB/FhaC/HecB family hemolysin secretion/activation protein | Resistance Mechanisms and Cell Survival |  |  |  |
| 9 | snp | CDS | missense_variant c.688C>A p.Leu230Ile | narX_1 | nitrate/nitrite two-component system sensor histidine kinase NarX | Resistance Mechanisms and Cell Survival |  |  |  |
| 10 | snp | CDS | missense_variant c.568T>C p.Phe190Leu | macB_1 | macrolide ABC transporter ATP-binding protein/permease MacB | Resistance Mechanisms and Cell Survival |  |  |  |
| 11 | snp | CDS | stop_gained c.1652C>G p.Ser551* | cas3 | CRISPR-associated nuclease/helicase Cas3 subtypeI-F/YPEST | Resistance Mechanisms and Cell Survival |  |  |  |
| 12 | snp | CDS | missense_variant c.172G>A p.Ala58Thr | arsB_1 | arsenite/antimonite:H(+) antiporter ArsB | Resistance Mechanisms and Cell Survival |  |  |  |
| 13 | snp | CDS | synonymous_variant c.321C>T p.Ala107Ala | arsD_2 | Arsenical resistance operon trans-acting repressor ArsD | Resistance Mechanisms and Cell Survival |  |  |  |
| 14 | snp | CDS | synonymous_variant c.876G>A p.Arg292Arg | rdgC_2 | Recombination-associated protein RdgC | Protein Synthesis and Genetic Information Processing |  |  |  |
| 15 | snp | CDS | missense_variant c.219G>A p.Met73Ile | MurR/RpiR | MurR/RpiR family transcriptional regulator | Protein Synthesis and Genetic Information Processing |  |  |  |
| 16 | snp | CDS | missense_variant c.670G>A p.Gly224Ser | recD | exodeoxyribonuclease V subunit alpha | Protein Synthesis and Genetic Information Processing |  |  |  |
| 17 | snp | CDS | missense_variant c.634A>G p.Thr212Ala | dnaX | DNA polymerase III subunit gamma/tau | Protein Synthesis and Genetic Information Processing |  |  |  |
| 18 | snp | CDS | missense_variant c.605A>C p.His202Pro | rluA | bifunctional tRNA pseudouridine(32) synthase/23SrRNA pseudouridine(746) synthase RluA | Protein Synthesis and Genetic Information Processing |  |  |  |
| 19 | snp | CDS | synonymous_variant c.459C>T p.Val153Val | rpsC | 30S ribosomal protein S3 | Protein Synthesis and Genetic Information Processing |  |  |  |
| 20 | snp | CDS | missense_variant c.379A>C p.Thr127Pro | xylB | xylulokinase | Metabolism |  |  |  |
| 21 | snp | CDS | missense_variant c.418A>G p.Thr140Ala | btuD_3 | Vitamin B12 import ATP-binding protein BtuD | Metabolism |  |  |  |
| 22 | snp | CDS | stop_gained c.481C>T p.Gln161* | torA | trimethylamine-N-oxide reductase TorA | Metabolism |  |  |  |
| 23 | snp | CDS | missense_variant c.172A>G p.Thr58Ala | pyk | pyruvate kinase | Metabolism |  |  |  |
| 24 | snp | CDS | missense_variant c.550A>T p.Ser184Cys | fre_2 | NAD(P)H-flavin reductase | Metabolism |  |  |  |
| 25 | snp | CDS | missense_variant c.1243G>A p.Asp415Asn | gadB | glutamate decarboxylase | Metabolism |  |  |  |
| 26 | snp | CDS | missense_variant c.466T>C p.Ser156Pro | purT | formate-dependent phosphoribosylglycinamide formyltransferase | Metabolism |  |  |  |
| 27 | del | CDS | frameshift_variant c.1978delG p.Ala660fs | fdnG_1 | formate dehydrogenase-N subunit alpha | Metabolism |  |  |  |
| 28 | snp | CDS | missense_variant c.821G>A p.Ser274Asn | dacA | D-alanyl-D-alanine carboxypeptidase DacA | Metabolism |  |  |  |
| 29 | snp | CDS | missense_variant c.76C>T p.Leu26Phe | caiC | crotonobetaine/carnitine-CoA ligase | Metabolism |  |  |  |
| 30 | snp | CDS | synonymous_variant c.270G>A p.Leu90Leu | dadX | catabolic alanine racemase DadX | Metabolism |  |  |  |
| 31 | snp | CDS | missense_variant c.101G>A p.Gly34Glu | amino acid permease | amino acid permease | Metabolism |  |  |  |
| 32 | snp | CDS | missense_variant c.476T>C p.Phe159Ser | alpha-keto acid decarboxylase family protein | alpha-keto acid decarboxylase family protein | Metabolism |  |  |  |
| 33 | snp | CDS | missense_variant c.266T>C p.Leu89Pro | yfdR | 5'-deoxynucleotidase | Metabolism |  |  |  |
| 34 | snp | CDS | missense_variant c.452G>A p.Gly151Asp | ompC | porin OmpC | Cellular Structure and Transport |  |  |  |
| 35 | ins | CDS | conservative_inframe_insertion c.1258_1260dupGGG p.Gly420dup | ftsI | peptidoglycan glycosyltransferase FtsI | Cellular Structure and Transport |  |  |  |
| 36 | snp | CDS | missense_variant c.1064A>C p.Asp355Ala | mrdA | peptidoglycan DD-transpeptidase MrdA | Cellular Structure and Transport |  |  |  |
| 37 | snp | CDS | missense_variant c.83G>A p.Ser28Asn | lptF | LPS export ABC transporter permease LptF | Cellular Structure and Transport |  |  |  |
| 38 | snp | CDS | missense_variant c.290T>C p.Val97Ala | pitA | inorganic phosphate transporter PitA | Cellular Structure and Transport |  |  |  |
| 39 | del | CDS | frameshift_variant c.1426delG p.Ala476fs | pitA | inorganic phosphate transporter PitA | Cellular Structure and Transport |  |  |  |
| 40 | snp | CDS | missense_variant c.782A>C p.Lys261Thr | FliC/FljB | FliC/FljB family flagellin | Cellular Structure and Transport |  |  |  |
| 41 | snp | CDS | missense_variant c.652A>G p.Ser218Gly | FliC/FljB | FliC/FljB family flagellin | Cellular Structure and Transport |  |  |  |
| 42 | snp | CDS | missense_variant c.1459G>A p.Gly487Arg | mukB | chromosome partition protein MukB | Cellular Structure and Transport |  |  |  |
| 43 | snp | CDS | missense_variant c.821G>A p.Gly274Glu | tamA | autotransporter assembly complex protein TamA | Cellular Structure and Transport |  |  |  |
| 44 | snp | CDS | missense_variant c.142A>G p.Asn48Asp | dcuA_2 | anaerobic C4-dicarboxylate transporter DcuA | Cellular Structure and Transport |  |  |  |
|  |  |  |  |  |  |  |  |  |  |
|  |  |  |  |  |  |  |  |  |  |
|  |  |  |  |  |  |  |  |  |  |
| **Isolate B3** | |  |  |  |  |  |  |  |  |
| S/N | TYPE | FTYPE | EFFECT | GENE | PRODUCT | CATEGORY |  |  |  |
| 1 | snp | CDS | missense_variant c.229C>T p.Leu77Phe | YgiW/YdeI | YgiW/YdeI family stress tolerance OB fold protein | Stress Response and Regulatory Functions |  |  |  |
| 2 | snp | CDS | missense_variant c.46G>A p.Ala16Thr | dpiA | two-component response regulator DpiA | Stress Response and Regulatory Functions |  |  |  |
| 3 | snp | CDS | missense_variant c.688C>A p.Leu230Ile | narX_1 | nitrate/nitrite two-component system sensor histidine kinase NarX | Stress Response and Regulatory Functions |  |  |  |
| 4 | snp | CDS | missense_variant c.308T>A p.Leu103Gln | ynaI | low conductance mechanosensitive channel YnaI | Stress Response and Regulatory Functions |  |  |  |
| 5 | snp | CDS | stop_gained c.1652C>G p.Ser551* | cas3 | CRISPR-associated nuclease/helicase Cas3 subtypeI-F/YPEST | Stress Response and Regulatory Functions |  |  |  |
| 6 | snp | CDS | missense_variant c.278C>A p.Thr93Lys | alkylhydroperoxidase domain protein | alkylhydroperoxidase domain protein | Stress Response and Regulatory Functions |  |  |  |
| 7 | snp | CDS | synonymous_variant c.180G>C p.Gly60Gly | AAA family ATPase | AAA family ATPase | Stress Response and Regulatory Functions |  |  |  |
| 8 | snp | CDS | missense_variant c.568T>C p.Phe190Leu | macB_1 | macrolide ABC transporter ATP-binding protein/permease MacB | Resistance Mechanisms and Cell Survival |  |  |  |
| 9 | snp | CDS | missense_variant c.821G>A p.Ser274Asn | dacA | D-alanyl-D-alanine carboxypeptidase DacA | Resistance Mechanisms and Cell Survival |  |  |  |
| 10 | snp | CDS | missense_variant c.172G>A p.Ala58Thr | arsB_1 | arsenite/antimonite:H(+) antiporter ArsB | Resistance Mechanisms and Cell Survival |  |  |  |
| 11 | snp | CDS | synonymous_variant c.321C>T p.Ala107Ala | arsD_2 | Arsenical resistance operon trans-acting repressor ArsD | Resistance Mechanisms and Cell Survival |  |  |  |
| 12 | snp | CDS | synonymous_variant c.876G>A p.Arg292Arg | rdgC_2 | Recombination-associated protein RdgC | Protein Synthesis and Genetic Information Processing |  |  |  |
| 13 | snp | CDS | missense_variant c.219G>A p.Met73Ile | MurR/RpiR | MurR/RpiR family transcriptional regulator | Protein Synthesis and Genetic Information Processing |  |  |  |
| 14 | snp | CDS | missense_variant c.334G>A p.Ala112Thr | purR | HTH-type transcriptional repressor PurR | Protein Synthesis and Genetic Information Processing |  |  |  |
| 15 | snp | CDS | missense_variant c.670G>A p.Gly224Ser | recD | exodeoxyribonuclease V subunit alpha | Protein Synthesis and Genetic Information Processing |  |  |  |
| 16 | snp | CDS | missense_variant c.634A>G p.Thr212Ala | dnaX | DNA polymerase III subunit gamma/tau | Protein Synthesis and Genetic Information Processing |  |  |  |
| 17 | snp | CDS | missense_variant c.1459G>A p.Gly487Arg | mukB | chromosome partition protein MukB | Protein Synthesis and Genetic Information Processing |  |  |  |
| 18 | snp | CDS | synonymous_variant c.459C>T p.Val153Val | rpsC | 30S ribosomal protein S3 | Protein Synthesis and Genetic Information Processing |  |  |  |
| 19 | snp | CDS | missense_variant c.379A>C p.Thr127Pro | xylB | xylulokinase | Metabolism |  |  |  |
| 20 | snp | CDS | missense_variant c.418A>G p.Thr140Ala | btuD_3 | Vitamin B12 import ATP-binding protein BtuD | Metabolism |  |  |  |
| 21 | snp | CDS | stop_gained c.481C>T p.Gln161* | torA | trimethylamine-N-oxide reductase TorA | Metabolism |  |  |  |
| 22 | snp | CDS | missense_variant c.172A>G p.Thr58Ala | pyk | pyruvate kinase | Metabolism |  |  |  |
| 23 | snp | CDS | missense_variant c.550A>T p.Ser184Cys | fre_2 | NAD(P)H-flavin reductase | Metabolism |  |  |  |
| 24 | snp | CDS | missense_variant c.290T>C p.Val97Ala | pitA | inorganic phosphate transporter PitA | Metabolism |  |  |  |
| 25 | del | CDS | frameshift_variant c.1426delG p.Ala476fs | pitA | inorganic phosphate transporter PitA | Metabolism |  |  |  |
| 26 | del | CDS | frameshift_variant c.96delG p.Trp32fs | glutathionylspermidine synthase family protein | glutathionylspermidine synthase family protein | Metabolism |  |  |  |
| 27 | snp | CDS | missense_variant c.1243G>A p.Asp415Asn | gadB | glutamate decarboxylase | Metabolism |  |  |  |
| 28 | snp | CDS | missense_variant c.466T>C p.Ser156Pro | purT | formate-dependent phosphoribosylglycinamide formyltransferase | Metabolism |  |  |  |
| 29 | del | CDS | frameshift_variant c.1978delG p.Ala660fs | fdnG_1 | formate dehydrogenase-N subunit alpha | Metabolism |  |  |  |
| 30 | snp | CDS | missense_variant c.76C>T p.Leu26Phe | caiC | crotonobetaine/carnitine-CoA ligase | Metabolism |  |  |  |
| 31 | snp | CDS | synonymous_variant c.270G>A p.Leu90Leu | dadX | catabolic alanine racemase DadX | Metabolism |  |  |  |
| 32 | snp | CDS | missense_variant c.142A>G p.Asn48Asp | dcuA_2 | anaerobic C4-dicarboxylate transporter DcuA | Metabolism |  |  |  |
| 33 | snp | CDS | missense_variant c.101G>A p.Gly34Glu | amino acid permease | amino acid permease | Metabolism |  |  |  |
| 34 | snp | CDS | missense_variant c.476T>C p.Phe159Ser | alpha-keto acid decarboxylase family protein | alpha-keto acid decarboxylase family protein | Metabolism |  |  |  |
| 35 | snp | CDS | missense_variant c.266T>C p.Leu89Pro | yfdR | 5'-deoxynucleotidase | Metabolism |  |  |  |
| 36 | snp | CDS | missense_variant c.1348T>A p.Cys450Ser | vgrG | type VI secretion system tip protein VgrG | Cellular Structure and Transport |  |  |  |
| 37 | del | CDS | frameshift_variant c.1498delC p.His500fs | yddB | TonB-dependent receptor plug domain-containing protein | Cellular Structure and Transport |  |  |  |
| 38 | snp | CDS | missense_variant c.91T>A p.Ser31Thr | ShlB/FhaC/HecB | ShlB/FhaC/HecB family hemolysin secretion/activation protein | Cellular Structure and Transport |  |  |  |
| 39 | snp | CDS | missense_variant c.452G>A p.Gly151Asp | ompC | porin OmpC | Cellular Structure and Transport |  |  |  |
| 40 | ins | CDS | conservative_inframe_insertion c.1258_1260dupGGG p.Gly420dup | ftsI | peptidoglycan glycosyltransferase FtsI | Cellular Structure and Transport |  |  |  |
| 41 | snp | CDS | missense_variant c.1064A>C p.Asp355Ala | mrdA | peptidoglycan DD-transpeptidase MrdA | Cellular Structure and Transport |  |  |  |
| 42 | snp | CDS | missense_variant c.83G>A p.Ser28Asn | lptF | LPS export ABC transporter permease LptF | Cellular Structure and Transport |  |  |  |
| 43 | snp | CDS | missense_variant c.782A>C p.Lys261Thr | FliC/FljB | FliC/FljB family flagellin | Cellular Structure and Transport |  |  |  |
| 44 | snp | CDS | missense_variant c.652A>G p.Ser218Gly | FliC/FljB | FliC/FljB family flagellin | Cellular Structure and Transport |  |  |  |
| 45 | snp | CDS | missense_variant c.821G>A p.Gly274Glu | tamA | autotransporter assembly complex protein TamA | Cellular Structure and Transport |  |  |  |


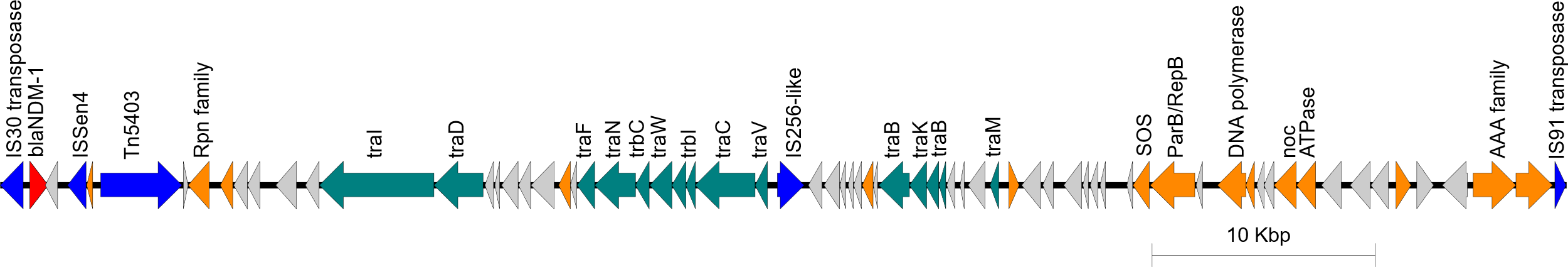


71,501bp


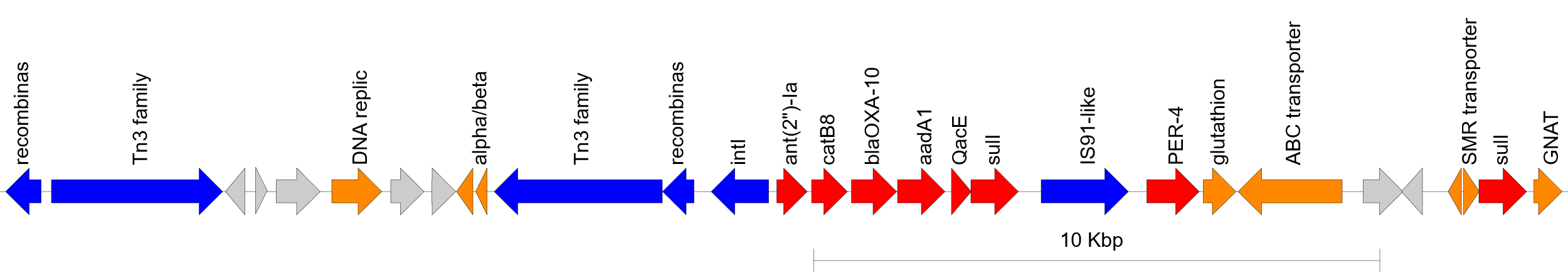


27,712bp


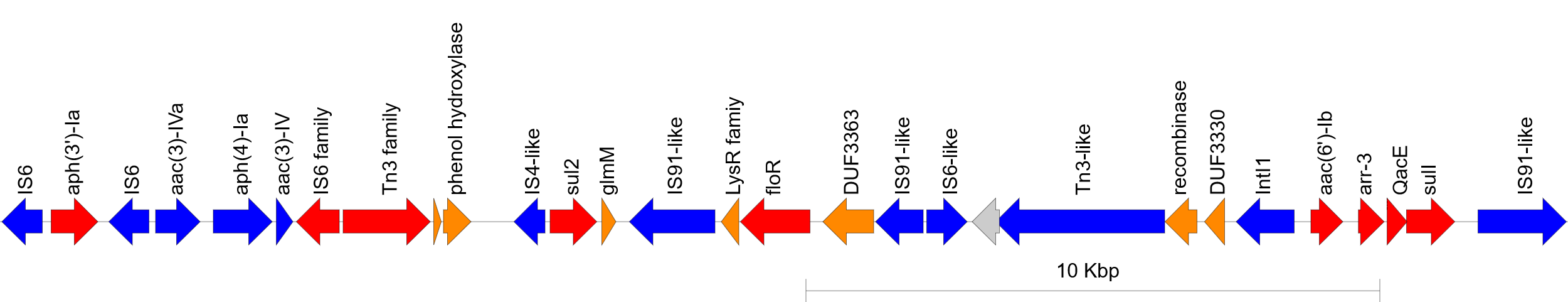


27,330bp

**Supplementary Figure 1.** Plasmid contigs bearing acquired antimicrobial resistance genes.
Contigs were assembled from the representative index isolate R1; identical contigs were also identified in the sequential isolates. Coding sequences (CDSs) are depicted as arrows indicating transcriptional direction. Red arrows denote antimicrobial resistance (AMR) genes; blue arrows indicate transposable elements such as insertion sequences and transposases; green arrows represent conjugative or mobilizable elements involved in plasmid transfer; orange arrows correspond to other functional genes; and light grey arrows represent hypothetical proteins. The length of each assembled contig is indicated.
